# National Inventory of Core Capabilities for Pandemic Influenza Preparedness and Response: Results from 36 countries with reviews in 2008 and 2010

**DOI:** 10.1111/irv.12214

**Published:** 2013-12-02

**Authors:** Ann Moen, Pamela J Kennedy, Po-Yung Cheng, Goldie MacDonald

**Affiliations:** a Influenza Division, Centers for Disease Control and PreventionAtlanta, GA, USA; b McKing ConsultingAtlanta, GA, USA; c BattelleAtlanta, GA, USA; d Center for Global Health, Centers for Disease Control and PreventionAtlanta, GA, USA

**Keywords:** influenza, monitoring and evaluation, pandemic preparedness

## Abstract

**Background:**

Re-emergence in 2003 of human cases of avian H5N1 and the resultant spread of the disease highlighted the need to improve the capacity of countries to detect and contain novel viruses. To assess development in this capacity, the Centers for Disease Control and Prevention (CDC) produced a tool for assessing a country's capability in 12 critical areas related to pandemic preparedness, including monitoring and identifying novel influenza viruses.

**Objectives:**

Capabilities the CDC tool assesses range from how well a country has planned and is prepared for an outbreak to how prepared a country is to respond when a pandemic occurs. Included in this assessment tool are questions to determine whether a country has a detailed preparedness plan and the laboratory capacity to identify various strains of influenza quickly and accurately.

**Methods:**

The tool was used first in 2008 when 40 countries in collaboration with CDC calculated baseline scores and used a second time in 2010 by 36 of the original 40 countries to determine whether they had improved their preparedness. Using basic mathematical comparison and statistical analyses, we compared data at the aggregate capability level as well as at the indicator and country levels. Additionally, we examined the comments of respondents to the assessment questionnaire for reasons (positive and negative) that would explain changes in scores from 2008 to 2010.

**Results:**

Analysis of results of two assessments in 36 countries shows statistically significant improvement in all 12 capabilities on an aggregate level and 47 of 50 indicators.

## Introduction

Re-emergence of human cases of avian H5N1 in 2003 spurred global efforts to assist countries to develop or increase their capacity to detect and contain novel influenza viruses. In September 2004, the United States Centers for Disease Control and Prevention (CDC) announced a program that would assist countries to develop their surveillance capacity. CDC entered into bilateral cooperative agreements with nine countries that identified cases of H5N1 or were close to such a country, adding an additional three countries in 2005. The purpose of the agreements was to improve surveillance, epidemiology, and laboratory infrastructure related to influenza detection. In addition to funding, the cooperative agreements provided countries with training and technical assistance to address important gaps in infrastructure and surveillance. Countries were eligible to enter into this CDC bilateral agreement only if they had an established national influenza center (NIC) with terms of reference agreeing to share relevant data and samples with the WHO Global Influenza Surveillance and Response System (GISRS).

When H5N1 spread to a peak of 115 human cases in nine countries in 2006^1^, the US government released the National Strategy for Pandemic Influenza (national strategy)^2^, identifying three pillars of activity and investment: 1) preparedness and communication, 2) surveillance and detection, and 3) response and containment. In accordance with the national strategy, CDC expanded its bilateral program for pandemic preparedness and response to include 29 additional countries globally. Specifically, the bilateral agreements provided CDC funding and technical support in developing infrastructure and capacity relevant to laboratory diagnostics, epidemiology, and surveillance; rapid response teams; national preparedness plans; communication strategies for healthcare workers; and related activities. Although expanding the WHO GISRS and assisting countries to meet the terms for designation as a NIC remained a priority, countries were no longer required to have a NIC. Countries without a NIC used the funds awarded to work toward fulfilling requirements for NIC designation.^3^ In addition to supporting individual countries, CDC established cooperative agreements with the WHO and all six of its regional offices to support improvements in surveillance and pandemic preparedness. CDC project officers were assigned to each recipient country to guide and support activities. In 2006, CDC began developing a tool to assess and document countries' capabilities in preparing for and responding to pandemic influenza. The objectives were to measure changes in each country's capacity over time and to assess how the US government and other global partners' financial and technical assistance were improving preparedness.^4^ At the time, few tools existed that could measure progress in building capacity for pandemic preparedness. The WHO checklist, which recorded the absence or presence of essential minimum elements of preparedness in a national plan to determine completeness of pandemic plans^5^, was frequently used. Rather than document planning elements such as the WHO checklist, CDC recognized the need for a tool that could assess discrete progress over time, with an increase in score linked to tangible evidence of improved systems. The CDC-produced assessment tool is the National Inventory of Core Capabilities for Pandemic Influenza Preparedness and Response (national inventory tool). Tool design took two years, and the design methodology is published concurrently with this study.^6^ The national inventory tool was designed to be administered periodically to gauge a country's progress toward having capacities important for dealing with an influenza pandemic. This article looks at scores of 36 countries that conducted the assessment twice: once to establish a baseline in 2008 and again approximately 2 years later to measure improvements.

## Methods

In 2008, CDC used the national inventory tool^7^ to document the status of 40 partner countries' preparedness for an influenza pandemic. CDC instructed each country in use of the tool, including recommendations on participants in the assessment and the value of including all sectors involved in pandemic preparedness and response within the country. CDC provided the assessment tool in advance to ensure that individuals and teams with the requisite content expertise or knowledge of the status of preparedness would be included in implementing the assessment. CDC worked with the host country to plan and conduct facilitated assessments using the national inventory tool. Two people from CDC participated in each assessment in 2008; one served as co-facilitator with the host country's representative, and the other documented the discussion and results. In some cases, additional CDC staff from the host country participated in discussions.

To ensure consistency in data collection across sites, CDC relied on three facilitators for most 2008 assessments. All CDC staff participating in assessments received training on use of the tool. In each country, facilitators guided participants through the tool, focusing on one indicator at a time and discussing the question that each indicator was meant to address.^6^ If there were difficulties in understanding the indicator, the facilitators used measurement notes included in the tool for clarification. Measurement notes provide definitions for terms in the indicator and examples of documentation and references. Assessment participants discussed each of 50 indicators in 12 capabilities. Facilitators asked a series of questions to reach score consensus and recorded a score of 0, 1, 2, or 3 for each indicator, documenting the discussions with notes to show how the final score was reached. The scores correspond to specific milestones that must be reached on the path to improved preparedness and response capability. A score of 0 denotes little or no demonstration of the function represented by the indicator, and a score of 3 denotes advanced accomplishment relevant to the indicator. Upon completion, the facilitator shared a draft report of scores and comments with the country's participants for validation. In 2010, 36 countries completed a second assessment; the average number of days between assessments was 694, with a range of 601 to 820 days. In 2010, usually one CDC staff member participated in facilitation and data collection along with a host national co-facilitator and relevant in-country staff. Many in-country assessors were the same in 2008 and 2010. The 2008 assessments established baseline scores at the indicator and capability levels and an estimate of each country's overall level of preparedness. Of the 36 countries repeating the assessment in 2010, 7 are low income, 28 are mid-income, and 1 is high income according to the World Bank designations.^8^

Each country's 2010 score was compared with its 2008 score. We recorded an increase in score from 2008 to 2010 as positive movement, a decrease as negative movement, and the same score in both years as no change. In addition to comparing scores using a simple percentage analysis, we made a statistical comparison at the capability and indicator levels. We used the Wilcoxon's signed-rank test to determine statistically significant changes in scores from 2008 to 2010. We performed the test on scores for each country and in aggregate by capability and indicator. A *P* value of <0·05 indicated statistical significance. To rank the relative performance of each capability or indicator, we first compared the *P* values. The smaller the *P* value, the higher the rank (1 being the highest rank). As so many *P* values were <0·0001, we compared the statistics from the Wilcoxon's signed-rank test: The higher the statistic obtained, the higher the rank order. We used SAS 9·1·3 for all analyses.^9^ Finally, we looked for any patterns of positive or negative movement by specific indicator, country, and income status.

## Results

On the basis of a nonparametric signed-rank test, when all 36 countries are considered in aggregate, all 12 capabilities showed statistically significant improvement from 2008 to 2010 (*P* value<0·0001 for 11 of 12 capabilities) (Table 1). Epidemiologic capacity had a *P* value of 0·0098. Table 2 shows the capabilities and indicators with a short description of the content of the indicator. For the 36 countries that participated in assessments in 2008 and 2010, Table 2 compares the aggregate average scores for each year and indicator. The number of countries with positive, no change, or negative movement in score is shown. A nonparametric signed-rank test of indicators showed statistically significant improvements for 47 of 50 indicators from 2008 to 2010, with *P* values <0·0001 for 28 of the 47 indicators. The three indicators lacking statistical significance were number of practicing epidemiologists in the population (4B), quality of practicing epidemiologists (4C), and availability of infection control materials (11C). As 11 capabilities and so many indicators had a *P* value of <0·0001, a statistical method to examine (or order) the indicators was applied to determine the relative performance of each capability or indicator. The rank order of the capabilities (Table 1) and indicators (Table 2) is shown in the last column. From 2008 to 2010, indicator scores showed positive movement 53·97% of the time, showed no change 38·70% of the time, and negative movement 7·33% of the time. Thirty-three of the 36 countries showed a decrease in scores for one or more indicators, with three countries (8%) reporting a decrease for more than 10 indicators. Only three countries recorded either positive movement or no change for all indicators.

**Table 1:** Signed-rank test comparing aggregate scores from 36 countries for each capability between 2008 and 2010

Capability	Capability name	Signed-rank test (nonparametric method)		Rank Order
		*P* value	Statistic	
1	Country Planning	<0·0001	229·5	8
2	Research and Use of Findings for Pandemic Influenza Preparedness	<0·0001	261·5	5
3	Communications	<0·0001	307	2
4	Epidemiologic Capability	0·0098	120·5	12
5	Laboratory Capability	<0·0001	196	10
6	Routine Influenza Surveillance	<0·0001	288·5	3
7	National Respiratory Disease Surveillance and Reporting	<0·0001	249·5	7
8	Outbreak Response	<0·0001	145	11
9	Resources for Containment	<0·0001	315	1
10	Community-Based Interventions to Prevent The Spread of Influenza	<0·0001	278	4
11	Infection Control	<0·0001	213	9
12	Health Sector Pandemic Response	<0·0001	259	6

**Table 2:** Comparison of aggregate indicator scores between 2008 and 2010

Capability name	Capability/Indicator	Description (Scoring range)	AVG 2008 score	AVG 2010 score	No. of countries (movement)			Signed-rank test (nonparametric method)		Rank Order
					Positive	None	Negative	*P* value	Statistic	
Country Planning	1A	Status of Plan	1·72	2·5	22	13	1	<0·0001	129·5	19
	1B	Dissemination	1·28	2·17	19	14	3	<0·0001	110	22
	1C	Exercises	1·36	2·19	21	14	1	<0·0001	110	22
	1D	Coordination	1·53	2·31	20	11	5	<0·0001	125	20
	1D1	Resources	1·08	1·68	19	12	3	<0·0001	99·5	28
Research and Use of Findings for Pandemic Influenza Preparedness	2A	Collaboration	1·14	1·5	17	14	5	0·0369	59	46
	2B	Research Priorities	0·75	1·39	15	18	3	0·0010	70·5	32
	2C	Environment of Support	1·03	1·61	17	16	3	0·0009	82·5	31
	2D	Use of data	0·92	2·25	28	7	1	<0·0001	210	3
Communications	3A	Status of Communications Plan	1·67	2·31	18	15	3	0·0026	78·5	36
	3B	Messaging	1·33	2·5	24	8	4	<0·0001	175	7
	3C	Dissemination	1·31	2·39	26	9	1	<0·0001	180·5	6
	3D	Staffing	1·08	2·19	24	10	2	<0·0001	161·5	10
Epidemiologic Capability	4A	Operational status	1·44	1·89	14	18	4	0·0261	50	43
	4B	Epidemiologists	1·56	1·81	12	19	5	0·1749	29·5	49
	4C	Quality	1·31	1·58	14	14	8	0·2286	36·5	50
	4D	Training	2·0	2·42	15	17	4	0·0076	63	38
Laboratory Capability	5A	Laboratory Network	1·83	2·53	16	18	2	0·0013	69·5	35
	5B	Bio-safety Level	1·83	2·08	11	22	3	0·0479	31·5	47
	5C	Methods	1·50	1·83	13	21	2	0·0065	45	37
	5D	Participation in WHO system	1·56	2·25	18	18	0	<0·0001	85·5	29
Routine Influenza Surveillance	6A	Integrated Surveillance	1·36	2·44	25	10	1	<0·0001	167·5	8
	6B	Data publication	1·44	2·58	24	11	1	<0·0001	154·5	11
	6C	Timeliness	1·33	2·42	25	9	2	<0·0001	163	9
	6D	Case definitions	2·67	2·94	9	25	2	0·0361	24	45
National Respiratory Disease Surveillance and Reporting	7A	Awareness of need to report	1·28	2·22	26	5	5	<0·0001	198	5
	7B	Rumor reporting	0·97	1·36	19	10	7	0·0231	81	42
	7C	Cross-notification	1·42	2·14	20	14	2	<0·0001	102	26
	7D	Timeliness	1·56	2·39	22	11	3	<0·0001	135·5	16
Outbreak Response	8A	Human resources	1·94	2·36	14	18	4	0·0089	57·5	39
	8B	Logistical resources	1·36	2·42	23	12	1	<0·0001	143·5	12
	8C	Exercises	1·5	2·28	19	13	4	0·0003	104	25
	8D	Activation of team	1·63	2·54	14	13	2	0·0011	59	34
Resources for Containment	9A	Availability of antivirals	0·89	2·09	23	12	0	<0·0001	138	15
	9B	Storage facilities	1·83	2·14	11	23	2	0·0164	33·5	40
	9C	Exercises	0·64	2·14	28	7	1	<0·0001	211·5	2
	9D	Distribution of materials	1·89	2·25	12	22	2	0·0220	35·5	41
Community-Based Interventions to Prevent The Spread of Influenza	10A	Social distancing	0·78	1·72	23	12	1	<0·0001	143	13
	10B	Critical infrastructure	0·53	1·28	23	12	1	<0·0001	140	14
	10C	Voluntary Isolation	0·94	2·25	32	2	2	<0·0001	277·5	1
	10D	Percent of Districts with plan	0·36	1·44	22	12	2	<0·0001	130	18
Infection Control	11A	Standards of Infection control	0·97	1·53	17	17	2	0·0011	81	34
	11B	Human resources	1·00	1·97	22	13	1	<0·0001	131	17
	11C	Logistical resources	1·00	1·39	17	10	9	0·0993	63	48
	11D	Institutionalization of Infection control	1·08	1·5	11	22	3	0·0317	34·5	44
Health Sector Pandemic Response	12A	Surge Capacity Human resources	0·11	0·97	22	13	1	<0·0001	120·5	21
	12B	Surge Capacity Facilities	0·36	1·03	19	17	0	<0·0001	85·5	29
	12B1	Surge Capacity Facilities	0·31	0·94	15	19	0	<0·0001	60	30
	12C	Clinical guidelines	1·08	2·72	28	7	1	<0·0001	205	4
	12D	Surge Capacity Care of Deceased	0·28	0·89	19	13	4	0·0003	102	26

In addition to analyzing the data in aggregate, we performed a signed-rank test on scores for individual countries. Of 36 countries that participated in both assessments, 30 made statistically significant progress, with a *P* value of 0·001 in 14 of 30 countries.

For data analyzed in aggregate, the overall trend was an increase in scores. Analysis of data at the country level for trends in specific indicators showed seven indicators with decreased scoring for five or more countries from 2008 to 2010 (Table 2, Movement). Conversely, five or more countries showed large increases in score (from 0 to 3) for 9 indicators from 2008 to 2010 (Table 3). Only one country reported a large decrease (from 3 to 0) for one indicator relevant to communications. Lastly, at 0·75 points, the increase in overall average score from 2008 to 2010 for 7 low-income countries (1·08 to 1·83) and 28 mid-income countries (1·30 to 2·05) was the same although mid-income countries started with a higher average baseline.^8^

**Table 3:** Number of Countries with indicator scores moving from 0 to 3 for each capability between 2008 and 2010

Capability	Capability name	Indicator					
		A	B	B1	C	D	D1
1	Country Planning	0	4		2	2	0
2	Research and Use of Findings for Pandemic Influenza Preparedness	1	2		1	6	
3	Communications	2	7		3	5	
4	Epidemiologic Capability	3	0		2	0	
5	Laboratory Capability	2	0		0	0	
6	Routine Influenza Surveillance	4	8		5	2	
7	National Respiratory Disease Surveillance and Reporting	1	1		1	3	
8	Outbreak Response	0	4		3	5	
9	Resources for Containment	2	0		9	0	
10	Community-Based Interventions to Prevent The Spread of Influenza	1	0		2	8	
11	Infection Control	1	4		2	2	
12	Health Sector Pandemic Response	3	1	0	12	1	

The number of raw scores possible for 2008 and 2010 was 3600. We have a total of 3588 scores, with 12 scores missing (8 for 2008 and 4 for 2010). Two scores for indicator 12B1 and six for the indicator 8D are missing for 2008. For 2010, one for 8D, two for 1D1, and one for 9A are missing.

## Discussion

The 2009 A (H1N1) pdm pandemic accelerated the development of some core capabilities. Although all capabilities showed statistically significant progress from 2008 to 2010, the capabilities with the greatest increase deserve further examination. Table 1 shows that the largest increases were, in order, in capabilities 9 (resources for containment), 3 (communications), 6 (routine influenza surveillance), and 10 (community-based interventions). It is important to look at the indicators relevant to these capabilities. Using the calculated statistic for *P* values <0·0001, we ranked indicators from most significant to least significant performance (Table 2). Many indicators with the greatest increase from 2008 to 2010 represent activities or functions that lend themselves to rapid improvements with concerted effort by the country or with donated goods from the international community. For example, as needs became evident during the pandemic, countries quickly addressed issues related to voluntary isolation and quarantine (10C), exercising or practicing for containment (9C), using data to inform decisions for pandemic preparedness (2D), and awareness of the need to report A (H1N1) pdm (7A). Other indicators with the greatest increases in scores represent activities or functions that improved rapidly because the country developed capacity or put infrastructure in place to prepare for and respond prior to the A (H1N1) pandemic. Those indicators are 12C: implementation of clinical guidelines; 3C: testing of formal and informal channels of communication; 3B: development and testing of communication materials; 6A: sentinel sites collecting virologic and epidemiologic data; and 6C: timeliness of reporting influenza surveillance data.

The 10 indicators that improved most from 2008 to 2010 (Table 2) proved their value under pandemic conditions. For example, surveillance systems and laboratory methods established to handle an H5N1 outbreak allowed countries to respond to the 2009 A (H1N1) pdm pandemic effectively. Likewise, because countries developed teams and practiced response to H5N1 outbreaks, they were able to respond quickly to the A (H1N1) pdm outbreak. Similarly, because countries had set up communications systems in preparation for H5N1, they were able to produce timely reports on the status of the outbreak. Only three indicators did not show statistical significance from 2008 to 2010: 4B and 4C (epidemiologic capacity) and 11C (infection control). The epidemiologic indicators relate to the presence and competency of field or practicing epidemiologists within a country. In general, the scores for these indicators remained the same from 2008 to 2010, highlighting how slowly factors related to human resources improve. Little or no change in the indicator of infection control shows that infection control materials at hospitals did not improve from 2008 to 2010.

Improvements for several of the indicators with the least progress from 2008 to 2010 (Table 2) would have required substantial investment of resources and time (e.g., availability of practicing public health epidemiologists). Two of the indicators were related to improving facilities (i.e., increasing bio-safety levels for public health laboratories and creating climate-controlled storage for materials). Improving these indicators requires considerable time and dedicated resources that may be cost-prohibitive for some low-resource countries. Collaboration between human and agricultural health authorities showed only modest improvements in scores from 2008 to 2010. Similarly, the use of standard case definitions for surveillance did not improve substantially, largely because most countries scored a 2 or 3 in 2008, leaving little room for improvement. That some indicators showed little progress from 2008 to 2010 was not unexpected given the long time needed to develop human resources or to improve buildings or facilities. Lack of improvement in human resources highlights the need to establish or sustain activities such as field epidemiology training programs to ensure that countries have appropriate human resources to handle disease outbreaks.

We examined changes in scores from 2008 to 2010 for each country, looking for trends in specific indicators. The movement columns of Table 2 show that scores for seven indicators decreased for five or more countries from 2008 to 2010. Three of these (4B, 4C, and 11C) are indicators that did not have statistically significant changes from 2008 to 2010. Two of the seven (2A: collaboration between human and animal health authorities; and 7B: monitoring rumors) are among the 10 indicators with the lowest improvement in score. The other two indicators with a negative trend in five or more countries were 1D1: mechanisms for sustainability of financial resources for pandemic planning; and 7A: awareness of the need to report by mounting public awareness campaigns. Although dedicated resources for preparedness and response activities improved greatly in some countries, in other countries the lack of resources for these activities persisted from 2008 to 2010. The decline in scores relevant to public awareness of the need to report pandemic H1N1 was likely related to the countries' health authorities placing less emphasis on the urgent need to report H1N1 than on the need to report H5N1.

Five or more countries reported improvements for 9 indicators. Of those 9 indicators, 7 are ranked among the top 10 in Table 2. Most of these indicators represent activities or functions for which substantial improvements can be made quickly, if the proper foundation is already in place. Additionally, the H1N1 pandemic forced many countries with little or no activity in some areas to rapid capacity advancement. For example, they were forced to set up systems to disseminate plans, use research findings to develop recommendations, create communication materials, train spokespersons, improve the timeliness of surveillance activities, and activate teams for outbreak investigations. Many countries' efforts to control a pandemic, in combination with systems established through preparedness efforts, resulted in large positive changes in indicator scores.

In addition to analyzing scores, we also analyzed the notes taken during the 2008 and 2010 assessments. We learned through this analysis that although channels of communication were established and functioning in many countries, the actual messages pertaining to A (H1N1) pdm were sometimes slow to be developed. Participants highlighted the importance of developing draft materials in advance of a pandemic and the value of other countries or partner organizations sharing materials.

Although the 2009 pandemic resulted in improvements in certain capabilities, some scores seem to have decreased because of the pandemic. Many countries with scores lower in 2010 than in 2008 said that before the pandemic they were confident of their capabilities because of their planning and preparation. However, their perceptions about their level of capability changed when faced with the realities of a pandemic. Therefore, some countries reported a lower score on an indicator after the opportunity to test their function in pandemic conditions. In particular, their efforts to put into effect community-based interventions to prevent the spread of influenza (capability 10) gave them an opportunity to test the effectiveness of their plans.

The six countries that did not have statistically significant improvements from 2008 to 2010 were distributed globally, with no more than two countries in any WHO region. Of the six countries, two had average overall scores higher than 2 (2·01 and 2·13) in 2008 and two had scores in the upper range of overall average scores (1·81 and 1·85). So, although their improvements were not statistically significant, four of these six countries had relatively high average baseline scores.

For the 12 missing responses for 2008 and 2010 combined, 8D (activation and deployment of trained, equipped teams in response to a public health emergency of international concern[PHEIC]) accounted for 58% of missing scores: six countries had no score in 2008 and one in 2010. This finding could relate to the newness of the International Health Regulations (IHR) language and the lack of a need to respond to a PHEIC before the 2008 assessment. Of the other missing scores, three involved absolute counts of beds or antivirals. We found when an indicator was assessed through absolute numbers, it was harder for countries to determine status. Two of the non-scored indicators in 2010 dealt with whether a country had a clearly defined decision-making structure in place, something identified by at least two of the countries as an issue during the 2009 pandemic.

The assessment tool has some limitations. CDC facilitators made every effort to standardize data collection in 2008 and 2010 and maintain consistency in conducting assessments between countries and over time. However, the final score was always determined by the country. Scores for some indicators may be inflated. However, according to CDC facilitators working with countries on these assessments, this did not seem to be the case.

## Conclusions

**Figure 1:**
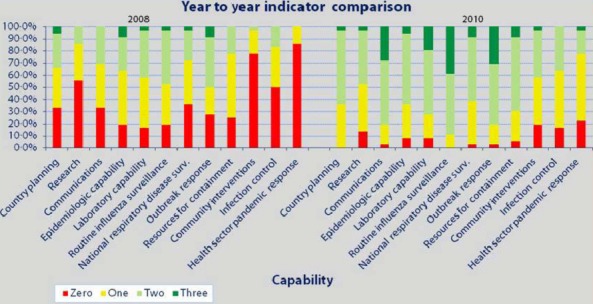
Aggregate year-to-year comparison of indicators for 36 countries.

CDC's national inventory tool shows that rigorous assessment of progress toward dealing with complex public health issues such as pandemic preparedness is possible. CDC created and used this tool for two main purposes. The first was to measure the effect on pandemic preparedness of providing technical assistance and support to partner countries. Figure 1 shows the aggregate capacity scores for 36 countries through the CDC assessment tool in both 2008 and 2010 where red represents an average score of 0–0·99, yellow 1–<2, light green 2–<3, and dark green an average of 3. This figure indicates that partner countries are making progress and that CDC and other international agencies providing support are having a positive effect on preparedness and response to pandemics. The second purpose was to learn how CDC could assist countries to fill gaps in their preparedness for pandemics by offering technical expertise in laboratory, epidemiology, and outbreak response. Countries have shown that they can use the results of the assessments to spur improvement in areas of need.^10^ Moreover, the capacity for pandemic response built through technical assistance from CDC, other agencies, and the countries' own work and that documented through these assessments contributes to increased worldwide capacity to follow IHR and serves countries in their work beyond influenza preparedness and response.
